# Factors impacting treatment and recovery in Anorexia Nervosa: qualitative findings from an online questionnaire

**DOI:** 10.1186/s40337-016-0107-1

**Published:** 2016-05-18

**Authors:** Sarah Fogarty, Lucie M. Ramjan

**Affiliations:** School of Medicine, Western Sydney University, Richmond, Australia; National Institute of Complementary Medicine, Western Sydney University, PO Box 2002, Homebush West, NSW 2140 Australia; School of Nursing and Midwifery, Western Sydney University, Richmond, Australia; Centre for Applied Nursing Research, Ingham Institute of Applied Medical Research, Richmond, Australia

**Keywords:** Anorexia nervosa, Treatment, Recovery

## Abstract

**Background:**

Anorexia nervosa (AN) is characterised by restriction of energy intake, fear of gaining weight and severe disturbances in weight or shape. Recovery from AN is a complicated and often multifaceted experience that can take many years to achieve. Qualitative research has found that support, being understood, hope, desire for recovery, positive experiences in treatment, self-efficacy, motivation and relationships are important in recovery from AN. The experience of treatment for patients with an eating disorder is an important aspect of recovery with the potential to enhance recovery or hinder it. The aim of the questionnaire was to better understand factors impacting the care experiences during treatment and or recovery from self-reported Anorexia Nervosa (AN).

**Method:**

An online questionnaire was developed and administered to past or current sufferers of Anorexia Nervosa, ≥18 years of age. Participants were recruited through eating disorder organisations both in Australia and the United Kingdom. The questionnaire was a mixture of quantitative and qualitative questions. The quantitative data was analysed using descriptive statistics and the qualitative data was analysed using conventional content analysis (CCA).

**Results:**

Of those who responded, most currently experienced self-reported AN. The quantitative results identified that most participants had trust and confidence in their health care provider and felt listened to and supported yet on the subject of the suitability of treatment this had varied opinions. Being understood, hope (life after AN) and self-acceptance were considered the top three important factors in the treatment and recovery from Anorexia Nervosa. The qualitative results revealed the factors hindering or benefiting treatment and recovery, and individuals’ needs during the four phases of recovery.

**Conclusion:**

Factors were identified that could either hinder or benefit treatment and recovery and these included whether treatment supported the individual to cope with change, whether the individual found the treatment to be appropriate for their personal needs and whether treatment addressed underlying factors. Individuals’ needs differed during the four phases of recovery. The findings of the study may help treatment providers address key factors involved in recovery at the right stage of treatment however by the nature of the qualitative methodology conclusions are putative and further definitive research is indicated.

## Background

Anorexia nervosa (AN) is a mental illness which is characterised by restriction of energy intake, fear of gaining weight and severe disturbances in weight or shape [1]. Recovery from AN is a complicated and often multifaceted experience that can take many years to achieve [2]. Research into recovery has commonly employed a qualitative methodology however a small number of researchers have used quantitative methods to explore recovery from AN. Support, being understood, hope, desire for recovery, positive experiences in treatment, self-efficacy, motivation and relationships are the quantitative themes that have been identified as important in recovery from AN [3–8].

The experience of treatment for patients with an eating disorder is an important aspect of recovery with the potential to enhance recovery [3] or hinder it [9]. Patients diagnosed with AN entering a treatment program may encounter difficulties including facing the challenges of changing unhealthy eating behaviors [10, 11]. The challenges of changing unhealthy eating behaviors is often complicated by patients displaying an ambivalence to making changes related to eating behaviors [10, 11]. Long-term exposure to eating disorder pathology and repeated failed attempts at reducing eating disorder symptoms may lead to an increase in ambivalence and a sense of hopelessness (from the patient as well as the clinician/therapist perspective), creating even more barriers to attaining healthy eating behaviors. Recent research has identified and categorised four phases involved in the recovery process; Unable and/or unready to change, Tipping point of change, Active pursuit of recovery and, Reflection and rehabilitation [4]. Given the influence of the treatment experience in recovery, understanding what is considered reliable and efficient treatment, is particularly important. Understanding the needs of AN sufferers during the different phases of recovery is an emerging area of research and a more in-depth understanding of AN patients’ experience of care in each of these phases is needed. Using an explorative qualitative methodology the paper aimed to better understand the care experience during treatment for AN in individuals with self-reported AN or recovery from AN”.

## Method

The treatment experiences questionnaire specifically examined the therapeutic relationship and care experience during treatment for AN. Ethical approval was obtained from the University of Western Sydney’s Human Research and Ethics Committee (HREC), H10825.

### Questionnaire development

The authors developed an anonymous questionnaire, which consisted of 30-items and, included 14 closed questions and 16 open-ended questions. The open-ended items encouraged written responses on individuals’ personal experience of care during the treatment of AN. Questions were included to elicit responses to the therapeutic relationships encountered and the care received during treatment for AN. Recent research revealed there are four phases in the recovery process: Unable and/or unready to change, Tipping point of change, Active pursuit of recovery and, Reflection and Rehabilitation [4]. There were a number of questions in the questionnaire that elicited a response about care encountered during these four stages.

The questionnaire used a purposive sampling technique which aims to recruit those who have particular expertise in the relevant topic of interest [12]. The sample in this case was recovered or recovering individuals with AN.

### Patient sample and procedure

The survey was completed online. A number of eating disorder associations including The Butterfly Foundation, BEAT UK (Beat Eating Disorders) and the Centre of Excellence for Eating Disorders advertised the study and provided a link to the online questionnaire. Sites advertised the study for a period of nine months. The questionnaire was anonymous. Inclusion criteria included being 18 years of age or over and having self-reported AN or having recovered from self-reported AN. Exclusion criteria included not being able to read and write in English. One hundred and sixty-eight individuals with self-reported AN consented to participate in the online questionnaire. Of the 168, seven agreed to participate but did not complete any questions thus 161 participants completed a combination of some to all of the questions. The 46.6 % of participants that answered the gender and age questions, were all women, average age 25.8 years ± 7.0 and the majority were students. One responder mentioned they were male but did not answer the gender question. No responder identified as an Aboriginal or Torres Straight Islander. See Table 1 for demographic details.

**Table 1:** Demographics of participants

Demographic Questions	Completed	% Answered
Completed	68 (*n* = 142)	47.89
Age		
Mean	25.11	
SD	6.44	
eOccupation		
Full time student	20	41.18
Employed	27	44.11
Other ^a^	5	7.35
Place of Birth	*n* = 67	
Australia	29	
United Kingdom	26	
Other ^b^	12	

^a^ part time student/employed, homemaker, unemployed

^b^ Scotland, Wales, Northern Ireland, United States of America, Canada, New Zealand, Italy, Africa, Germany, Portugal

Accessing participants in both the UK and Australia was chosen to increase study participant numbers, taking into consideration ethics and similarities in treatments. The ‘standard’ treatment offered for Anorexia Nervosa is similar with both the UK and Australia utilising a multidimensional treatment approach, addressing the physical, psychological, psychosocial and family needs of the individual. Treatment can include psychiatrists, psychologists, primary care physicians, social workers, nurses and dieticians [13, 14]. In general both countries have similar treatment aims; to restore the individual’s weight within a normal range for their height and age, to reduce abnormal eating behaviours, and weight and shape cognitions, and manage co-morbidities (both mental and physical) [13, 14]. In both the UK and Australia we were able to provide contact details for support services should any study participants experience distress.

### Data analysis

Descriptive statistics were used to analyse demographic data such as overall sample size, demographic characteristics (location, age) and the responses to the closed questions. Data were analysed using Microsoft Excel for Mac, version 14.4.7.

The responses to the open-ended questions was analysed using a *Conventional Content Analysis* (CCA) technique [15]. The CCA approach allows the analysis (categories and names for the categories) to be derived directly from the text data, rather than being preconceived [15]. The purpose of CCA is to identify patterns in textual material. CCA is a dynamic form of textual analysis which summarises the informational content of data [16].

The first author read all the survey responses multiple times to achieve immersion [15]. Responses were then read in depth and frequently repeated words (e.g. understanding) denoting main views on treatment and recovery were manually circled on hard copy data extracts. First impressions of data were noted and formed the basis of development of ‘Sub Categories’ for grouping into main ‘Categories’ [15]. A quasi-statistical analysis style was used to determine a hierarchical summary of the ‘important features of recovery and treatment’ based on the frequency of views expressed [16]. Associations and relationships within sub categories and categories were identified and where relevant they were combined into a smaller number of sub categories and categories which reflected the perceptions of the individuals with AN [15]. The first author coded and analysed all questionnaire data. Results were checked by the second author to determine if categories and subcategories were mutual. Any differences were discussed until consensus was reached.

## Results

Sixty-eight (42.5 %) of the responders currently had self-reported AN and over 97 % received some form of treatment (either inpatient or outpatient care or a combination of both inpatient and outpatient care or another version of care) (See Table 2). The majority or responders felt they were treated well (*n* = 64), able to trust and had confidence in their treatment providers (*n* = 93) and felt that their treatment providers listened and understood their issues (*n* = 100). The appropriateness of the treatment program was mixed with some responders feeling that treatment was suitable (*n* = 55) and others feeling it did not suit them (*n* = 63). The majority of responders felt they were unready and or unable to change when they sought treatment (*n* = 62). Being understood, hope (possibility of life after AN), and self-acceptance were all viewed as important factors in the treatment of AN.

**Table 2:** Quantitative Results

Question 1. How long ago did you have Anorexia Nervosa (AN)? (*n* = 154, 100.00 %)		%
Have it now	63	40.91
Less than 12 months ago	20	12.99
12 months to less than 2 years ago	11	7.14
2 years to less than 5 years ago	23	14.94
5 years to less than 10 years ago	20	12.99
Greater than 10 years ago	15	9.74
Other	2	1.30
Question 2. What type of treatment did you receive for your AN? (*n* = 153, 99.35 %)		%
Combination of both (in and out patient)	77	50.33
In-patient	14	9.15
Outpatient	53	34.64
Other	9	5.88
Question 3a. How well were you treated when you first sought treatment? (*n* = 133, 86.36 %)		%
I was treated well	64	48.12
I was not treated so well	57	42.86
No opinion	12	9.02
Question 3b. How suitable is/was the treatment program for you? (*n* = 133, 86.36 %)		
It has suited me	55	41.35
It did not suit me	63	47.37
No opinion	15	11.28
Question 3c. Do you think your treatment providers have been able to listen and understand the things you have brought up during treatment? (*n* = 133, 86.36 %)		
Yes, to a great extent	33	25.00
Yes to some extent	67	50.76
No	32	24.24
Question 3d. Did/Do you feel trust and confidence in the treatment providers? (*n* = 133, 86.36 %)		
Yes, to a great extent	33	24.81
Yes to some extent	60	45.11
No, not sufficiently	40	30.08
Question 3e. Are/Were you and the treatment providers in agreement about the goals of your treatment? (*n* = 132, 85.71 %)		
Yes, to a great extent	30	22.72
Yes to some extent	72	54.55
No	30	22.72
Question 4. Please indicate which phase you believed you were in when you sought treatment (multiple answers allowed) (*n* = 93, 60.39 %)		
Unready and or unable to change	62	67.39
The Tipping point of change	37	43.53
Active pursuit of recovery	26	30.59
Reflection and rehabilitation	5	5.88
Question 7. Research has identified key themes of recovery factors in AN. Please indicate which factors you feel are important in the treatment of AN (multiple answers allowed) (*n* = 93, 60.39 %)		
Being Understood	71	76.34
Self-discovery	44	47.31
Hope (possibility of change)	63	67.74
Hope (possibility of life after AN)	68	73.12
Self-acceptance	64	68.82
Improved self-esteem	55	59.14
Engagement with therapist or treatment provider	62	66.67
None	1	1.08

### Results of the open-ended questions

The open-ended questions addressed two main areas: factors hindering or benefiting treatment and recovery and identifying what was needed during the four phases of recovery.

The three themes that were seen to both hinder and contribute to recovery were: *1*) *supporting a person to cope with change* (*subthemes of hope*, *being listened to and understood*, *and empowerment*/*disempowerment*), *2*) *treatment suitability and*, *3*) *treat underlying factors*.

### Supporting a person to cope with change

The treatment experience impacted on the individuals’ perception of support with treatment either providing support for the patient to change and benefiting recovery or not providing support for change and disempowering the individual and hindering recovery. The elements of treatment that contributed to participants experiencing support included hopefulness, being listened to and understood, having input into recovery, increased self-worth and learning new skills and coping mechanisms. These elements contributed to make the individuals with AN feel stronger and more confident, especially in controlling their AN and facing the challenges that ‘recovery’ entails.

The elements of treatment that contributed to participants feeling unsupported included not being heard, or understood, control taken away, being infantised, threats and punishments. These elements largely attributed to the individuals with AN feeling unsafe, incapable and undeserving of being able to face the challenges that ‘recovery’ entails.

### Subtheme: Hope

Imbuing hope through treatment helped individuals feel supported, normal and believed in and provided them with the motivation when confronted with challenges. A participant described positive experiences with her therapist: “[*she*] *invested her time and hope into me which made me feel worthy and deserving of life* (*112*)”. Hope that recovery was possible was either inherent and experienced by individuals with AN, as illustrated in the following quote: “*hope*, *the possibility of life after AN. We all want to know that we can beat this and that there is a life that doesn*’*t involve AN* (*63*)”. Alternatively it was nurtured through others such as a treatment provider: “*others having hope when I had lost all of mine* (*76*)”.

### Subtheme: Feeling listened to and understood

Feeling listened to and understood helped individuals to feel safe, validated and worthy and supported to open up and make changes. This is conveyed by the following participant quotes: *I trusted her 100* % (*a therapist*) *and she understood me so well*, *she made recovery feel more safe and achievable* (*60*)” and “*I feel understood*, *not alone in this horrible battle which makes me comfortable to be honest and open up about things I would find hard to discus with others due to embarrassment and fear of judgment* (*40*)”..

Not being heard and not being understood created an environment that was not conducive to coping with change: “*if you are misunderstood it*’*s hard to get to the cause of a problem and to find solutions or skills that a person is capable of implementing to overcome distress* (*84*)”. It also eroded trust in providers and lead to feelings of not being safe and unsupported during times of stress and change. For instance a participant explained that she did not feel understood, which “*made me feel I couldn*’*t be as honest about my ED behaviours* (*40*)” while another participant felt her treatment providers “*didn*’*t listen to how I really felt*, *didn*’*t care. Never once did I feel supported or cared for or safe* (*68*)”. Not being heard enforced feelings of not being ‘normal’ and feelings that “*the opinions about myself were wrong and stupid which left me feeling alienated* (*94*)”.

### Subtheme: Empowered/disempowered

The definition of empowerment is “to make (someone) stronger and more confident, especially in controlling their life and claiming their rights” [17]. Individuals with AN paradoxically display control regarding the structure and regime of their exercise and eating yet they feel out of control by that same routine and structure [18] and the inability to change it [19]. Participants who had input into their recovery or some control over their recovery/treatment felt empowered by the changes they were making and their capacity to confront aspects of their AN. This was demonstrated by a participant who commented: “(*my treatment provider*) *allowed me to set the pace*, *meaning I felt a great sense of self*-*satisfaction as I took charge of my own recovery* (*75*)”. Consequently when individuals had more input into their own recovery their experience of treatment was positive: “*I have a great input into my own recovery which I believe has made it a better and more positive experience* (*142*)”. A sense of empowerment was also experienced as participants regained some control through learning new coping strategies and skills to help combat the disorder such as increasing self-worth and respect. This has been captured in the following participant quotes: “*I had a very low sense of self worth and a huge sense of failure*, *and it was very important to me that my therapist respected me as a person and not just an illness. I needed to feel they could see my strengths* (*88*)”, “*I was respected and taught the strategies for coping with emotions and difficult situations* (*79*)” and recovery was helped by “*improving self esteem*”. *We want to be able to look at our bodies*, *and smile when we are in public or looking in the mirror*, *we want to be confident and proud* (*63*)”.

When control was felt to be taken away from participants they experienced powerlessness and this heightened their sense of being ‘out of control’ which is intrinsic in AN [18]. This was illustrated by the following participants: “*I was forced to do things I could not cope with*, *the pace things were moving at was uncomfortable and it made me even worse as things were so out of my control* (*145*)” and “*when suffering from anorexia*, *control is a big thing. And having that taken away from you by professionals making choices on your behalf is very demeaning* (*68*)”.

A number of participants felt that they had been threatened and punished or treated like a child during their treatment for AN. Participants found this demoralising and damaging to their self-esteem. “*As patients it felt like we were treated like criminals*…. *We were punished regularly whenever we fell victim to our eating disorders and never encouraged for the times we were able to be strong and stand up to our distressing thoughts* (*75*)” One participant felt like she was “*constantly spoken to like a child or babied which actually fed my insecurities* (*68*)”. In many cases it was fear that motivated change rather than a desire to be recovered: “*I gained weight and did what I was told out of fear of being punished rather than because I felt supported to get well* (*41*)”.

### Treatment suitability

Treatment suitability was an important aspect in treatment and recovery. This theme included multiple domains, including access to treatment, treatment itself and the treatment providers. When treatment was deemed suitable to the person with AN, it was viewed as “*systematic*, *compassionate and individualised* (*46*)” and available regardless of weight gain and cost. For one participant it was having “*no time limit on treatment and being able to remain as inpatient at a healthy maintenance weight was crucial* (*96*)” which was crucial to recovery. When treatment was suitable individuals also felt supported by practitioners, friends and or family: “*having the knowledge that they were loved through their replaces*, *that they were safe to break down* (*19*)”. For some the non-clinical environment was “*warm and inviting*…*these things as well as the respectfulness of my treatment team made me feel safe and valued and my opinions respected* (*79*)”. Individuals often experienced treatment and the treatment providers as conveying “*empathy*, *patience*, *humour and a genuine belief in my worth as a person* (*82*)”.

When treatment was not suitable to the person with AN it was viewed as too rigid and not individualized such as having a “*one size fits all approach*-*if you don*’*t improve on their regime*, *you must not be trying hard enough* (*50*)”. Treatment was often, inaccessible, either due to cost or not being ill enough with participants experiencing “*being sent home until critical and in need of life saving* (*45*)” and trouble “*getting the GP to refer me to specialists as I was told my weight was not low enough* (*142*)”. Not being able to access treatment when needed and there is a genuine desire for help can lead to “*further distress*” and deterioration of health. One participant revealed that she struggled for three months to access treatment and “*by this point my BMI was so low I needed to be hospitalised. I believe*, *if I had support sooner*, *I would not have got that ill* (*104*)”. Treatment providers not having AN specific experience or skills negatively influenced recovery and the treatment experience with many participants experiencing treatment providers negatively: “*staff did not have a great knowledge or understanding about AN* (*28*)”. Treatment providers were often telling them to “*just eat*” which lead to participants feeling invalidated and helpless.

### Treat underlying factors

“*Eating disorders are not just about the weight on the scale* (*142*)” and when treatment was perceived to address more than just weight gain it was viewed as beneficial. Treating underlying factors and separating the eating disorder from self enabled a treatment that allowed individuals to focus on “*what the eating disorder represents*, *explore personality traits*, *interests and life* (*113*)”, and “*deal with the dysfunction underlying the disorder* (*140*)”. Treatment that “*addressed psychological*, *dietetic*, *medical and practical* (*97*)” aspects of the eating disorder was viewed as holistic. Treating underlying causes was also viewed as facilitating the conditions to “*learn new healthy coping strategies* (*105*, *140*)” and skills.

When treatment was perceived to address only weight then it was viewed as hindering recovery. Individuals felt strongly that there was a need to treat underlying factors, not just the signs/symptoms of AN. Individuals experienced a feeling of incomplete treatment, invalidation and lack of congruence about treatment and recovering. This occurred for individuals who were discharged at their ‘target weight’ and yet they still felt emotionally the same as explained by this participant: “*when in inpatient treatment the focus was on physical restoration so when I was discharged at the* ‘*target weight*’, *I was still the same emotionally as when I was admitted* (*141*)”. Some felt they had not learnt the skills they needed to cope during treatment: “*the goal was not to learn the coping skills necessary to overcome our illness*, *we were released as soon as we reached our* ‘*goal weight*’ (*75*)’”.

### The stages of recovery

The stages of recovery involved different experiences of treatment and different needs (See Table 3). The first stage of recovery (unready or unwilling to change) saw individuals feeling alone as they were thrust into a situation they were not ready for or didn’t understand. Support was important in stages 2-4 of recovery (tipping point of change, actively pursuing recovery and reflection and rehabilitation), but more important during the tipping point of change and in the reflection and rehabilitation stages. The right treatment and exceptional treatment providers were also important in stages 2-4 but more important in the active pursuit of recovery phase. Being understood was an important factor in the tipping point of change and self-directed recovery was important in the active pursuit of recovery phase.

**Table 3:** The phases of treatment and what impacted recovery

Four phases of AN treatment and what was experienced and or helped in each stage (ranked in order of importance)			
*Unable or unready to change*	*Tipping Point of Change*	*Actively pursuing recovery*	*Reflection and rehabilitation*
Forced into treatment	Supported (not alone)	Right treatment and great treatment providers	Support
Felt alone	Right treatment	Support	Right treatment & great treatment providers
Believed okay/didn’t have AN	Felt understood	Recovery self-directed (responsible for self)	Self-acceptance/personal worth/self love/kindness to self
Wasn’t ready to change	Involved in having a say in treatment	Set own goals	Encouraged
Wasn’t ready so rebelled	Recognised illness severity	More than AN (life outside AN)/Hope)	Skills to cope/manage
Wasn’t understood	Listened to		
Needed:	Trust		
More information (what to expect from treatment, how to manage AN)			
Treatment focusing on more than weight Support			
Time/space/therapy to recognise there was a problem			

## Discussion

This paper investigated the care experience during treatment for AN in a self-selected volunteer sample of patients with or recovered from AN and three themes were identified; supporting a person to cope with change, treatment suitability and treat underlying factors. These themes were found to be important and either hindered and/or benefited recovery during treatment.

In this study, the theme of supporting a person to cope with change involved the following subthemes: hopefulness, being listened to and understood, control in recovery, increased self-worth, learning new skills and coping mechanisms. Not supporting a person to cope with change involved not being listened to, not being understood, a lack of control in recovery and punishments, and threats. Treatments that emphasise and limit these factors might, therefore, be helpful in promoting recovery from AN. Our study augments the current research on the impact of support to change on the treatment experiences of individuals with AN and may provide valuable identification of some of the key factors that should align with treatment for recovery to occur. Empowerment has been shown to lead to better experiences of the quality of care received, better patient-provider communication, increased motivation and higher patient involvement in decision making [20, 21]. Motivation and empowerment have been linked to a more favourable treatment response for patients with AN including completing the course of treatment [4, 5, 8, 22–25]. The study findings of not being supported to change, lack of control and disempowerment align with current research, which shows that feeling disempowered and that treatment was likened to being in prison, hinders the development of the therapeutic relationship and has the potential to exacerbate ambivalence to change for those with AN [9, 11, 24, 26]. The importance of hope in recovery has been emphasised in previous research findings [3, 4, 27, 28] and our study emphasises the importance of hope in supporting change, recovery and the treatment experience. Although supporting patients is important and it might be one way of improving their perception and experience of care, which may have flow on effects for treatment efficacy and or compliance, it cannot wholly account or explain recovery. However focusing on the factors that promote individuals feeling supported might give treatment providers a piece of the puzzle. It may help in addressing the complex, multidimensional needs of individuals for recovery and keep patients engaged in treatment.

Treatment, which was viewed as suitable, involved individualised and accessible care with support during treatment however when treatment was deemed incompatible to the individual, it was too structured, not individualised and inaccessible. Difficulty accessing treatment for AN is not a new phenomenon with research identifying multiple causes including people not being ‘thin’ enough to access treatment [26]. Recovery has been shown to be influenced by intake BMI and age of first accessing treatment [26, 29], therefore it is imperative that individuals are able to access appropriate treatment without fear of denial or conditional prerequisites. The study findings draw attention to the importance of individualised treatment and are consistent with similar themes that characterise people with AN and their experience of treatment [24, 30–32]. These themes emphasise the struggle between the provision of effective AN treatment approaches and structure and the need for autonomy and a treatment structure that can meet the needs of the individuals. Consistent with earlier studies, treatment when experienced as incompatible to the individual, was felt to be too rigid and not tailored to the individuals’ needs [26, 31, 32] with patients experiencing oppression, and of being in a prison and restricted [9, 24, 25, 32]. Dissatisfaction with treatment has been linked with treatment inefficiency, delays in accessing treatments, increased risk of relapse, and premature cessation of treatment [25, 33–36]. The studies findings add to the factors that can be improved in the treatment of AN to make a considerable difference in recovery. A positive finding of this study was the important impact treatment providers can and are making by providing treatment that suits the individual. Despite this some individuals are not experiencing treatment that is applicable and thus more research is needed on how to improve the treatment experience for more individuals with AN. Quantitative methodologies should be employed to test hypotheses generated from the present study’s findings such as individualised treatment. For example for individualised treatment, a controlled trial employing programs that allow for individualised meal plans versus less flexible programs could be undertaken.

The importance of treating the underlying factors of AN, not just weight, is well established [26, 31, 37]. Our study findings reinforce the importance of treating underlying factors and provide an understanding of the factors that contribute to treatment being experienced as holistic. Treatment systems that have a strong emphasis on weight and food have been shown to have negative side effects including increased suicidal behaviour, restriction and reinforcing of AN behaviours [26, 38]. Being treated holistically has been identified as what is sought from AN treatments [26] but it is not clear what underlying factors require more emphasis and research. This study identifies treatment congruence with weight and life goals, the provision of coping and management skills, as important factors of a ‘holistic’ treatment and more research is needed on these factors such as a controlled trial program that employs a more ‘holistic’ treatment such as massage therapy or yoga versus more conventional treatments such as counseling.

Four phases or recovery have been identified and with that key factors associated with each phase [4]. The findings of this paper support initial research findings of not believing/understanding they had AN during the *unready* phase, feeling understood in the *tipping point of change* phase and self-acceptance in the *reflection* phase [3]. Our findings also support the theme of locus of control with the person moving from an external locus of control to an internal locus of control as recovery progresses. There were some original experiences found for each phase of recovery such as being forced into treatment and feeling alone during the *unready* phase, feeling supported and receiving the right treatment in the *tipping point of change*, the *active* and the *reflection* phases. Understanding how people with AN experience these phases might help treatment providers’ address the key concerns and feelings at the right stage of the treatment phase and has the potential to improve treatment experiences and therefore treatment outcomes. Further research addressing key factors and experiences during the different phases of recovery is warranted such as a controlled trial program that identifies AN patients in the unready to change phase and allocates them to an adjunct program that focused on being understood and not feeling alone versus an additional psychological treatment.

The study has several limitations. The participants included both recovered and non-recovered individuals and as such some of the participants’ experiences were retrospective. Participants were a self-selected volunteer sample with self-identified AN and as such were not representative of the diversity of people receiving treatment for AN. The study used qualitative exploratory methods and conclusions are thereby putative, not definitive. Future studies should investigate treatment experiences in representative samples such as clinical cohort studies with diagnostic and recovery status assessed by validated instruments. The time period of recover from AN for participants was not collected, in hindsight collection of this data should be have been collected. Another limitation was that participants were from two different geographic locations. However a strength of this study is the similarity of findings between both locations.

## Conclusion

This paper investigated the care experience during treatment for AN in a self-selected volunteer sample of patients with or recovered from AN and three themes emerged that identified factors that either hindered and/or benefited recovery; the effect of treatment on empowerment/disempowerment, treatment suitability and treat underlying factors. The stages of recovery involved different experiences of treatment and identified different needs during each stage which with further research may help treatment providers address key concerns at the right stage or in the right treatment phase of AN. Similarly to other qualitative research undertaken on the treatment experiences of those with AN, our study reiterates that many important interrelated factors are important in recovery however the nature of the qualitative methodology conclusions are putative and further definitive research is indicated.
